# Adaptive Seamless Phase II/III Randomization Test Considering Treatment Group Selection Based on Short–Term Binary Outcomes

**DOI:** 10.1002/sim.70400

**Published:** 2026-02-04

**Authors:** Funato Sato, Kohei Uemura, Junki Mizusawa, Yutaka Matsuyama, Yoshihiko Morikawa

**Affiliations:** ^1^ Research Promotion Center, Tokyo Metropolitan Hospital Organization Tokyo Japan; ^2^ Department of Biostatistics & Bioinformatics, Interfaculty Initiative in Information Studies The University of Tokyo Tokyo Japan; ^3^ Center for Research Administration and Support, National Cancer Center Tokyo Japan; ^4^ Department of Biostatistics School of Public Health, The University of Tokyo Tokyo Japan

**Keywords:** adaptive seamless phase II/III design, correlation, overall survival, randomization test, response rate, short–term outcome

## Abstract

Recently, adaptive seamless phase II/III designs (ASDs) have gained attention because they improve the efficiency of drug development. In an ASD, a phase II trial, which explores the dose–response relationships and identifies treatments for the phase III trial, is combined with a phase III trial, which aims to demonstrate the efficacy and safety of the selected treatment arms in a single trial. This study focused on ASD, which selects treatment groups based on the short–term outcomes observed early in the trial, and involves a confirmatory outcome as the long–term outcome. The method based on the combination test, which considers treatment group selection based on short–term outcomes, tends to be conservative. In other words, it controls the type I error rate more strictly than necessary as the correlation decreases among outcomes in phases II and III. To address this issue, we proposed an adaptive seamless phase II/III randomization test that can appropriately consider the correlation between outcomes based on a randomization distribution, where phase II has a binary outcome and phase III has overall survival. Based on the simulation study, the proposed method improved conservatism owing to the correlation among outcomes and controlled the type I error rate around the nominal level. In addition, the power of this method tended to be higher than that of the method based on the combination test in most scenarios. Overall, the proposed method can increase the probability of trial success compared with conventional phase III designs.

## 1 Introduction

In recent years, innovative clinical trial design methodologies that enable efficient drug development have garnered increasing attention [1]. Conventional clinical trials are conducted strictly according to a protocol defined before trial initiation. Even when adjustments to the pre–specified number of participants, treatment groups, or allocation ratios need to be modified during the course of the trial, deviations from the original plan are not permitted, which can reduce the probability of trial success. For example, the sample size pre–fixed in the protocol is typically calculated based on information available at the planning stage; however, such estimates are often based on uncertain information, such as the results of small trials. As a result, confirmatory trials may fail due to a lack of power [2].

An adaptive design is an innovative clinical trial design that allows changes to one or more facets of the pre–planned design, without compromising the validity or integrity of the trial. Such modifications are based on data accumulated from trial participants and are intended to increase the probability of trial success. The European and US regulatory authorities have issued mounting methodological research and held discussions on the implementation and guidance of adaptive designs [3, 4, 5]. In adaptive seamless phase II/III design (ASD), a trial starts with multiple treatments and control groups. At the interim analysis—corresponding to the end of phase II part of the trial—the dose–response relationship is explored, the optimal dose is determined, and the treatment group for phase III part is selected. The efficacy and safety of the selected treatment group are then verified in the final analysis, which corresponds to phase III part of the trial [6]. ASD allows phase II and III trials to be conducted as a single seamless clinical trial. Compared to conventional development strategies, ASD shortens development timelines by eliminating the period between the end of phase II and the initiation of the phase III trial. Additionally, by including data from phase II part in the final analysis, the total number of required participants can be reduced compared with the conventional, separate phase II and III trials. Furthermore, longer follow–up data can be obtained at the end of the trial [7].

The trial conducted by the Japan Clinical Oncology Group (JCOG2203), which served as the inspiration for this study, was a multicentre, randomized, controlled trial investigating the effect of combining preoperative chemotherapy with surgery plus adjuvant chemotherapy for resectable esophagogastric junction adenocarcinoma [8]. Two treatment regimens were evaluated: docetaxel + oxaliplatin + S–1 (DOS) and docetaxel + oxaliplatin + leucovorin + fluorouracil (FLOT). These regimens have demonstrated efficacy in previous phase III trials. However, no clinical trials have directly compared the two regimens. Through a questionnaire survey conducted by the research group, centers supporting each regimen were identified. As a result, it was deemed difficult to conduct a trial restricted to only one of the two regimens. Therefore, ASD was adopted to the JCOG2203 trial, in which one of the two treatment regimens was selected based on the pathological response rate in phase II part. Subsequently, the superiority of the selected treatment group over the standard treatment group was assessed based on overall survival in phase III part.

In the analysis of a trial formulated using ASD, it is essential to account for the inflation of the type I error rate that may arise when using data from the treatment group selection in the final analysis. Several statistical methods have been proposed to address this issue. Notably, methods based on group sequential design, combination tests, and conditional error functions can control the inflation of the type I error rate [7, 9, 10]. However, these methods assume that the same outcome is used for both treatment group selection and the final analysis. Further, in trials with a long follow–up period before primary outcome (long–term outcome) data are available, sufficient data are often lacking at the time of treatment group selection [11]. Therefore, the statistical methodology of ASD has been extended to allow treatment group selection based on a short–term outcome, which data can be accumulated within a shorter follow–up period [12, 13, 14, 15].

In the method proposed by Friede et al. [12], the actual family–wise error rate (FWER)—defined as the probability of rejecting at least one true null hypothesis under any constellation of null hypotheses—becomes smaller as the correlation between short– and long–term outcomes decreases. This results in a more conservative test, potentially leading to a loss of power and an increased sample size requirement. Takahashi et al. [16] investigated the mitigation of this conservatism in a setting where both the short– and long–term outcomes are binary, and proposed an exact test based on the conditional distribution of the test statistic in the final analysis given treatment group selection. However, when the outcome of phase III part is a survival variable, the correlation between the outcomes is difficult to account for explicitly. Moreover, no solution has been proposed for calculating the conditional distribution of the test statistics in the final analysis in such settings.

With reference to the JCOG2203 trial, we proposed a novel analytical method for ASD. This method appropriately accounts for the correlation between outcomes by using a randomization distribution based on randomization procedure when phase II part has a binary outcome and phase III part uses overall survival, which is commonly seen in oncology drug development. The performance of the proposed method was evaluated through simulation studies.

## 2 Methods

### 2.1 Assumed Clinical Trial Settings and Notation

In this study, we considered a two–stage ASD with treatment group selection, similar to the design used in the JCOG2203 trial. The short–term outcome used for the treatment group selection was a binary response to treatment, while the long–term outcome used for the final analysis was overall survival. The interim analysis, conducted at the end of Stage 1, did not involve any statistical tests for early stopping criteria for efficacy or futility.

In Stage 1, participants are randomly assigned to one of the G treatment group (g=1,2,…G) or a control group (g=0). The number of participants in each group is denoted by $N_{g,1}$ and the total number is given by $N_{1}=N_{0,1}+N_{1,1}+N_{2,1}+\cdot \cdot \cdot +N_{G,1}$. Let $X_{g}$ denote the number of responses in group g, which follow $\mathrm{Bin}\left(N_{g,1},\pi_{g}\right)$, where $\pi_{g}$ is the true response rate. The treatment group s with the highest response rate in the interim analysis, is conducted at the end of Stage 1, is selected to proceed to Stage 2. Here S is a random variable taking values of S=1,2,…G. If two or more treatment groups share the highest response rate (e.g., both treatment groups have a response rate of 0.5 when G=2), one group is randomly selected among them to select only one group for simplicity.

The participants included in Stage 2 are randomly assigned to either the selected treatment group or the control group. The number of participants in each group is denoted by $N_{g,2}\;\left(g=0,S\right)$, and the total number of participants in Stage 2 is given by $N_{2}=N_{0,2}+N_{S,2}$. In the final analysis, conducted at the end of Stage 2, the selected treatment is compared with the control group based on the long–term outcome obtained from all participants enrolled in treatment group S and the control group. Let, $\theta_{g}$ denote the true log hazard ratio for the comparison between the treatment group g and control group, where $\theta_{g}<0$ indicates that the treatment group g is superior to the control group. Let $u_{g,j},V_{g,j}$ represent the score statistic and Fisher information for the comparison between the treatment group g and control group at stage j(j=1,2), calculated using data obtained from $N_{g,j}$ which is the sample size of the treatment group g at stage j, respectively. The score statistic $u_{g,j}$ asymptotically follows a normal distribution with mean $\theta_{g}V_{g,j}$ and variance $V_{g,j}$. ${\hat{\theta }}_{g,j}$, defined as $u_{g,j}/V_{g,j}$, also asymptotically follows a normal distribution [17],

(1) $${\hat{\theta }}_{g,j}∼N\left(\theta_{g},\frac{1}{V_{g,j}}\right).$$

### 2.2 Combination Test Approach for Survival Outcome

The method proposed by Friede et al. [12] combines the long–term outcome data for each stage using a combination test. The approach for applying the combination test when the survival variable is the long–term outcome is described below.

Let each elementary null hypothesis be $H_{g}:\theta_{g}=0$ for g=1,2,…G. The standardized statistic $Z_{g,j}$ for comparing the treatment g and control group, based on the long–term outcome obtained in stage j, is expressed as $u_{g,j}/\sqrt{V_{g,j}}$, where $Z_{g,2}$ is calculated using only the Stage 2 data and not include accumulated data from Stage 1. The one–sided p–value is given by $p_{g,j}=1-\Phi \left(u_{g,j}/\sqrt{V_{g,j}}\right)$, where Φ(·) denotes the cumulative distribution function of the standard normal distribution.

To strictly control the FWER, a testing procedure that combines the closed testing procedure [18] with the combination test [19] method for paired comparisons with the control group is applied [12]. Let the intersection hypothesis be denoted by $H_{I}={⋂}_{g\in I}H_{g}\left(I\subseteq \left\{1,\ldots ,G\right\}\right)$. If all intersection hypotheses $H_{I}$, including the elementary null hypothesis $H_{S}$ for the treatment group S with the highest response rate in the interim analysis, are individually rejected at the one–sided level α in the final analysis, the null hypothesis of interest $H_{S}$ is also rejected at the one–sided level α, and the superiority of treatment group S over the control group is considered to be demonstrated. The test for each intersection hypotheses is constructed using a combination test that combines stagewise p–values calculated separately from the data at each stage. Let α denote the one–sided significance level, and let $\Phi^{-1}\left(\cdot \right)$ represent the inverse function of the cumulative distribution function of the standard normal distribution. Friede et al. used $p_{I,1}$ as the one–sided p–value for $H_{I}$ based on Stage 1 data, and $p_{I,2}$ as the corresponding p–value based on Stage 2 data. These values are then combined using the weighted inverse normal method [20]:

(2) $$\begin{array}{cc} & C\left(p_{I,1},p_{I,2}\right)=1-\Phi \left(w_{1}\Phi^{-1}\left(1-p_{I,1}\right)+w_{2}\Phi^{-1}\left(1-p_{I,2}\right)\right),\\ & \text{Reject}H_{I}\;\text{if}C\left(p_{I,1},p_{I,2}\right)\leq \alpha . \end{array}$$

The weights $w_{1}$ and $w_{2}$, which satisfy $0<w_{j}<1$ and $w_{1}^{2}+w_{2}^{2}=1$, are pre–specified and set proportional to the square root of the expected number of events at the final analysis in each stage. The Stage 1 p–value, $p_{I,1}$, is obtained by applying a Dunnett–type test [21] to control FWER based on the test statistic $Z_{I,1}^{\max}=\max_{i\in I}Z_{i,1}$. It should be noted that $Z_{I,1}^{\max}$ is not necessarily equal to $Z_{S,1}$, because treatment group S is selected based on the short–term outcome rather than the long–term outcome. In Stage 2, the comparison is performed between the treatment group S and the control group, so that $p_{I,2}=p_{g,2}$.

### 2.3 Adaptive Seamless Phase II/III Randomization Test in Case of Selecting One Treatment Group

The method of Friede et al. can be applied to control the FWER in ASD below the nominal level. However, as the correlation between short– and long–term outcomes decreases, the actual FWER tends to become smaller than the nominal level, making the testing procedure overly conservative [12]. This conservativism arises because the divergence increases between $Z_{I,1}^{\max}$, which serves as the multiplicity control in Stage 1, and $Z_{S,1}$, which actually contributes to the final analysis. Let $p_{I,1}=f\left[1-\Phi \left(Z_{I,1}^{\max}\right)\right]$, $p_{I,2}=p_{S,2}=1-\Phi \left(Z_{S,2}\right)$, and let f(.) be a function representing the penalty for calculating the adjusted p–value to perform Dunnett's test. For simplicity, we consider the case where the sample sizes $N_{1}$ and $N_{2}$ at each stage are sufficiently large:

(3) $$\begin{array}{c} \mathrm{Pr}\left[C\left(p_{I,1},p_{I,2}\right)\leq \alpha \right]\approx \alpha . \end{array}$$

Since both $p_{I,1}$ and $p_{S,2}$ asymptotically follow a uniform distribution under the null hypothesis, the asymptotic p–clud condition [22] is satisfied. Therefore, Equation (3) holds. However, as the correlation between short– and long–term outcomes decreases, the tendency for $Z_{S,1}<Z_{I,1}^{\max}$ becomes stronger. As a result, the actual FWER, which is the probability that the selected treatment group S shows a statistically significant difference in the final analysis, is given by $\mathrm{Pr}\left[C\left(p_{S,1},p_{S,2}\right)\leq \alpha \right]<\alpha$.

ALGORITHM 1 **Adaptive Seamless phase II/III randomization test in case of selecting one treatment group.**

1. Given the response and overall survival data of $N_{0,1},N_{1,1},\ldots ,N_{G,1}$ participants randomized in Stage 1, compute the observed test statistic $z_{k,1}^{*}$ when treatment group k is selected.
2. Rerandomize $N_{1}$ participants with the given response and overall survival data to groups with sizes $\left\{N_{0,1},N_{1,1},\ldots ,N_{G,1}\right\}$.
3. Given the above i–th rerandomized data, compute the rerandomized test statistic $Z_{k,1}^{\left(i\right)}$.
4. Repeat steps 2 and 3 for M iterations to obtain a set of test statistics $Z_{k,1}^{\left(1\right)},\ldots ,Z_{k,1}^{\left(i\right)},\ldots ,Z_{k,1}^{\left(M\right)}.$ If it is computationally infeasible to enumerate all possible randomizations, repeat steps 2 and 3 for $M^{*}$ times instead.
5. Compute the conditional p–value $p_{k\left|Q\left({\hat{\pi }}_{1}\right)\right.,1}$ using the empirical randomization distribution.

In this study, we propose an adaptive seamless phase II/III randomization test (ASRT) to address the conservatism of the FWER. Our method incorporates the approach proposed by Friede et al. to control multiplicity in ASD based on the closed test principle. Let ${\hat{\pi }}_{1}={\left({\hat{\pi }}_{1,1},{\hat{\pi }}_{2,1},\ldots ,{\hat{\pi }}_{G,1}\;\right)}^{T}$ denote a vector of observed response rates in the G treatment groups in Stage 1. Define $Q\left({\hat{\pi }}_{1}\right)$ as the selection function, where $Q\left({\hat{\pi }}_{1}\right)=k$ when treatment group k(k∈{1,…,G}) is selected on Stage 1 data,

(4) $$\begin{array}{c} Q\left({\hat{\pi }}_{1}\right)=k\;\text{if}\max\left({\hat{\pi }}_{1}\right)={\hat{\pi }}_{k,1}. \end{array}$$

For a multivariate probability distribution of the vector of test statistics ${\left(Z_{1,1},Z_{2,1},\ldots ,Z_{G,1}\right)}^{T}$ in Stage 1 based on long–term outcomes, and let c be an arbitrary constant, the marginal distribution of $Z_{S,1}$ is calculated as follows:

(5) $$\begin{array}{c} \mathrm{Pr}\left(Z_{S,1}\geq c\right)=\sum_{k=1}^{G}\mathrm{Pr}\left(Z_{k,1}\geq c,Q\left({\hat{\pi }}_{1}\right)=k\right). \end{array}$$

When comparing $Z_{g,1}$ of the randomly selected group g, the inequality $\mathrm{Pr}\left(Z_{k,1}\geq c,Q\left({\hat{\pi }}_{1}\right)=k\right)>\mathrm{Pr}\left(Z_{g,1}\geq c,Q\left({\hat{\pi }}_{1}\right)=k\right)$ holds due to the positive correlation between the selection function $Q\left({\hat{\pi }}_{1}\right)$ and $\arg_{g\in \left\{1,2,\ldots G\right\}}\max{\left(Z_{1,1},Z_{2,1},\ldots ,Z_{G,1}\right)}^{T}$. From this, the following conclusions can be drawn:

(6) $$\begin{array}{c} \mathrm{Pr}\left(Z_{S,1}\geq c\right)>\sum_{k=1}^{G}\mathrm{Pr}\left(Z_{g,1}\geq c,Q\left({\hat{\pi }}_{1}\right)=k\right)=\mathrm{Pr}\left(Z_{g,1}\geq c\right). \end{array}$$

The asymptotic p–clud condition [22] is satisfied for *p*–value $p_{g,1}=1-\Phi \left(Z_{g,1}\right)$ of a randomly selected group g, but not for the marginal *p*–value of selected group S, $p_{S,1}=1-\Phi \left(Z_{S,1}\right)$, as shown in Equation (6). Let $z_{k,1}^{*}$ denote the observed value of $Z_{k,1}$. To satisfy the asymptotic *p*–clud condition for $Z_{S,1}$, we consider the conditional distribution of $Z_{S,1}$ given the treatment group selection $Q\left({\hat{\pi }}_{1}\right)=k$ in Stage 1, which is similar to the method proposed by Takahashi et al. [16]:

(7) $$\begin{array}{c} \mathrm{Pr}\left(Z_{k,1}\geq z_{k,1}^{*}|Q\left({\hat{\pi }}_{1}\right)=k\right)=\frac{\mathrm{Pr}\left(Z_{k,1}\geq z_{k,1}^{*},Q\left({\hat{\pi }}_{1}\right)=k\right)}{\mathrm{Pr}\left(Q\left({\hat{\pi }}_{1}\right)=k\right)}. \end{array}$$

As in the postselection inference, selective type I error rate can be controlled by using 1 minus the distribution function conditional on selection, then the conditional probability $\mathrm{Pr}(Z_{k,1}\geq z_{k,1}^{*}|Q({\hat{\pi }}_{1})=k)$ denoted by $p_{k\left|Q({\hat{\pi }}_{1})\right.,1}$ follows an asymptotically uniform distribution under the null hypothesis $H_{k}$ [23, 24, 25]. For example, in case $Q({\hat{\pi }}_{1})$ coincides with 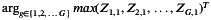, by symmetry considerations under $H_{k}$, pkQπ^1,1=PrZI,1max≥zk,1*1G1G=PrZI,1max≥zk,1*, and $\mathrm{Pr}\left(p_{k\left|Q\left({\hat{\pi }}_{1}\right)\right.,1}\leq \alpha \right)=\alpha$ in the long run for any 0≤α≤1 such as Lemma 3 in Tibshirani et al. [23]. By using $p_{k\left|Q\left({\hat{\pi }}_{1}\right)\right.,1}$ instead of the unconditional *p*–value calculated by $1-\Phi \left(z_{k,1}^{*}\right)$ ithe following combination test, based on this conditional *p*–value, enables control of the FWER in the ASD by satisfying the asymptotic p–clud condition [22]:

(8) $$\begin{array}{cc} & C\left(p_{k|Q\left({\hat{\pi }}_{1}\right),1},p_{k,2}\right)=1-\Phi \left(w_{1}\Phi^{-1}\left(1-p_{k|Q\left({\hat{\pi }}_{1}\right),1}\right)+w_{2}\Phi^{-1}\left(1-p_{k,2}\right)\right),\\ & \text{Reject}\;H_{k}\;\text{if}\;C\left(p_{k|Q\left({\hat{\pi }}_{1}\right),1},p_{k,2}\right)\leq \alpha . \end{array}$$

Takahashi et al. [16] calculated the conditional p–value, $p_{k\left|Q\left({\hat{\pi }}_{1}\right)\right.,1}$, using an exact method based on the binomial distribution, since the long–term outcome was also binary in their study. In contrast, the long–term outcome in this study is survival variable, making it difficult to obtain the conditional distribution given the short–term outcome. Therefore, we calculated $p_{k\left|Q\left({\hat{\pi }}_{1}\right)\right.,1}$ by applying the randomization test [26, 27] based on the randomization procedure. Using the response and overall survival data of $N_{0,1},N_{1,1},\ldots ,N_{G,1}$ participants randomized in Stage 1, we permuted only the allocation variables corresponding Stage 1 randomization. The total number of possible allocation patterns, M, is given as follows:

(9) $$\begin{array}{c} M=\left(\begin{array}{c} N_{1}\\ N_{0,1} \end{array}\right)\left(\begin{array}{c} N_{1}-N_{0,1}\\ N_{1,1} \end{array}\right)\ldots \left(\begin{array}{c} N_{G-1,1}-N_{G,1}\\ N_{G,1} \end{array}\right). \end{array}$$

Let ${\hat{\pi }}_{1}^{\left(i\right)}={\left({\hat{\pi }}_{0,1}^{\left(i\right)},{\hat{\pi }}_{1,1}^{\left(i\right)},\ldots ,{\hat{\pi }}_{G,1}^{\left(i\right)}\right)}^{T}$ denote the vector of response rates calculated based on the data of the $N_{0,1},N_{1,1},\ldots ,N_{G,1}$ participants under the i–th allocation pattern among the M possible allocation patterns. Let $Z_{k,1}^{\left(i\right)}$ be the test statistic for the treatment group k, calculated using the survival data corresponding to the i–th allocation. Then, the conditional p–value $p_{k\left|Q\left({\hat{\pi }}_{1}\right)\right.,1}$ can be calculated based on the randomization distribution as follows:

(10) $$\begin{array}{c} p_{k\left|Q\left({\hat{\pi }}_{1}\right)\right.,1}=\frac{\sum_{i=1}^{M}I\left[Z_{k,1}^{\left(i\right)}\geq z_{k,1}^{*}\right]\;I\left[Q\left({\hat{\pi }}_{1}^{\left(i\right)}\right)=k\right]}{\sum_{i=1}^{M}I\left[Q\left({\hat{\pi }}_{1}^{\left(i\right)}\right)=k\right]}, \end{array}$$

where I(·) is an indicator function that takes the value 1 if the condition inside the parentheses is true, and 0 otherwise. However, when $N_{1}$ is not extremely small, the calculation of $p_{k\left|Q\left({\hat{\pi }}_{1}\right)\right.,1}$ is often computationally infeasible. In such cases, the randomization distribution can be approximated by repeating the randomization procedure a sufficiently large number of times, denoted by $M^{*}$. The procedure for the ASRT is as follows (Algorithm 1):

### 2.4 Adaptive Seamless Phase II/III Randomization Test Allowing to Select More Than One Treatment Group

In this section, we expand the ASRT to allowing to select more than one treatment group. Let $\tilde{Q}\left({\hat{\pi }}_{1}\right)$ be the selection function that takes $\tilde{Q}\left({\hat{\pi }}_{1}\right)=l$ when treatment groups l(l⊆{1,…,G}) are selected. The selection function $\tilde{Q}\left({\hat{\pi }}_{1}\right)$ can be as follows:

(11) $$\begin{array}{c} \tilde{Q}\left({\hat{\pi }}_{1}\right)=l\;\text{if}\left|{\hat{\pi }}_{\forall m\in l,1}-\max\left({\hat{\pi }}_{1}\right)\right|\leq ∆, \end{array}$$

where ∆ is a margin considered to be clinically equivalent range of response rates. Let $p_{\tilde{k}\left|\tilde{Q}\left({\hat{\pi }}_{1}\right)\right.,1}=\mathrm{Pr}\left(Z_{\tilde{k},1}\geq z_{\tilde{k},1}^{*}|\tilde{Q}\left({\hat{\pi }}_{1}\right)=l\right)$ denote the conditional p–value of the each selected groups $\tilde{k}\in l$,$z_{\tilde{k},1}^{*}$ be the observed $Z_{\tilde{k},1}$ for the group $\tilde{k}$, in which $p_{\tilde{k}\left|\tilde{Q}\left({\hat{\pi }}_{1}\right)\right.,1}$, respectively, follows an asymptotically uniform distribution under the null hypothesis $H_{\tilde{k}}$. $p_{\tilde{k}\left|\tilde{Q}\left({\hat{\pi }}_{1}\right)\right.,1}$ can be calculated based on the randomization distribution as follows:

(12) $$\begin{array}{c} p_{\tilde{k}\left|\tilde{Q}\left({\hat{\pi }}_{1}\right)\right.,1}=\frac{\sum_{i=1}^{M}I\left[Z_{\tilde{k},1}^{\left(i\right)}\geq z_{\tilde{k},1}^{*}\right]\;I\left[\tilde{Q}\left({\hat{\pi }}_{1}^{\left(i\right)}\right)=l\right]}{\sum_{i=1}^{M}I\left[\tilde{Q}\left({\hat{\pi }}_{1}^{\left(i\right)}\right)=l\right]}. \end{array}$$

FWER of this ASD can be controlled by applying a multiple test method such as Bonferroni, Holm, and Dunnett methods to the following combination test *p*–values of the selected groups $C\left(p_{\tilde{k}\left|\tilde{Q}\left({\hat{\pi }}_{1}\right)\right.,1},p_{\tilde{k},2}\right)$, satisfying the asymptotic p–clud condition [22]:

(13) $$\begin{array}{c} C\left(p_{\tilde{k}\left|\tilde{Q}\left({\hat{\pi }}_{1}\right)\right.,1},p_{\tilde{k},2}\right)=1-\Phi \left(w_{1}\Phi^{-1}\left(1-p_{\tilde{k}\left|\tilde{Q}\left({\hat{\pi }}_{1}\right)\right.,1}\right)+w_{2}\Phi^{-1}\left(1-p_{\tilde{k},2}\right)\right),\tilde{k}\in l. \end{array}$$

## 3 Simulation Study

### 3.1 Simulation Experiment 1

To evaluate the performance of the proposed method and compare it with that of Friede et al., simulation studies were conducted under a two–stage ASD with treatment group selection, similar to the design used in the JCOG2203 trial. Simulation Experiment 1 assessed the actual type I error rate and power of both the proposed method and the method by Friede et al. The number of treatment groups was set to G=2 (g=1,2), and each was compared pairwise with a common control group (g=0). The sample sizes for each group at each stage were equal, $N_{g,1}=50$ and $N_{g,2}=150$. The enrolment periods for Stages 1 and 2 were 1.7 and 3.3 years, respectively, and the corresponding follow–up periods were 6.3 and 3 years. An interim analysis was performed 6 months after enrolment of $N_{g,1}$ patients in each group to select the treatment group for Stage 2. The treatment group with the highest observed response rate was selected to proceed to Stage 2, along with the control group. If multiple treatment groups showed tie response rate and shared the highest, one group is randomly selected among them. In the final analysis, the selected treatment group and the control group were compared based on the overall survival of 200 individuals. The weights $w_{1}$ and $w_{2}$ used in the combination test were calculated based on the expected number of events from $N_{g,1}$ and $N_{g,2}$ participants in the final analysis, $w_{1}=\sqrt{75/\left(75+179\right)}=0.543$ and $w_{2}=\sqrt{179/\left(75+179\right)}=0.839$. To adjust for the multiplicity arising from multiple group comparisons using the method of Friede et al., three correction methods were applied: the conservative and commonly used Bonferroni and Holm methods, as well as Dunnett method based on normal approximation.

#### 3.1.1 Data Generation

Correlated data for response rate and overall survival were generated using a copula approach, assuming a Clayton–type correlation structure for the bivariate survival variables: response time and overall survival [28] the joint survival function was defined as:

(14) $$\begin{array}{c} S\left(t_{g,R},t_{g,D}\right)={\left\{S_{g,R}{\left(t_{g,R}\right)}^{-\theta_{g}}+S_{g,D}{\left(t_{g,D}\right)}^{-\theta_{g}}-1\right\}}^{-1/\theta_{g}}, \end{array}$$

where $S_{g,R}\left(t_{g,R}\right)$ is the survival function for the nonnegative random variable $R_{g}$, representing the response time in group g, and $S_{g,D}\left(t_{g,D}\right)$ is the survival function for the overall survival $D_{g}$ in group g. Both $S_{g,R}\left(t_{g,R}\right)$ and $S_{g,D}\left(t_{g,D}\right)$ were assumed to follow exponential distributions with hazard rates $\lambda_{g,R}$ and $\lambda_{g,D}$, respectively. The parameter $\theta_{g}$ determines the strength of correlation between the response time $R_{g}$ and the overall survival $D_{g}$. Notably, the Kendall rank correlation coefficient $\tau_{g}$ is related to $\theta_{g}$ by the formula: $\tau_{g}=\theta_{g}/\left(\theta_{g}+2\right)$ [29]. Following convention, $\tau_{g}$—which is more interpretable than $\theta_{g}$—was used as the indicator of the correlation between response rate and overall survival. In this study, a common value of $\tau_{g}=\tau \in \left\{0.2,0.5,0.8\right\}$ was used for all groups. Response rate data were derived as a binary variable: a value of 1 was assigned if the response time exceeded 6 months, and 0 otherwise.

#### 3.1.2 Type I Error Rate

To evaluate the type I error rate, we established a global null hypothesis in which the true 3–year survival rates were set equally across all groups: $S_{0,D}\left(3\right)=S_{1,D}\left(3\right)=S_{2,D}\left(3\right)=0.5$. The true response rate was also set to be the same across all groups and varied as $S_{g,R}\left(0.5\right)\in \left\{0.4,0.5,0.6\right\}$. In the JCOG2203 trial, the significance level was loosely set at a one–sided 5%, reflecting the fact that the experimental treatments were provided in routine clinical practice and the enrolment of eligible patients was expected to be challenging. Accordingly, the significance level in this simulation was also set at a one–sided 5%. The type I error rate was defined as the proportion of rejecting the null hypothesis for a treatment group comparison based on overall survival. The number of simulation iterations was set to 10 000 and the *p*–values for the proposed method were computed approximately by repeating the randomization 5000 times.

#### 3.1.3 Power

Two scenarios were considered for power evaluation, as summarized in Table 1. In Scenario 1, only one treatment group was assumed to be effective, with $S_{1,D}\left(3\right)=0.6,S_{0,D}\left(3\right)=S_{2,D}\left(3\right)=0.5$. To evaluate the impact of response rate on power, and to assess how well the response rate enables the selection of a truly effective treatment based on overall survival, the response rate for treatment Group 1 was varied: $S_{1,R}\left(0.5\right)\in \left\{0.4,0.5,0.6\right\}$. A lower value of $S_{1,R}\left(0.5\right)=0.4$ indicates a more difficult scenario for correctly selecting treatment Group 1, whereas a higher value of $S_{1,R}\left(0.5\right)=0.6$ implies easier selection. The power in Scenario 1 was defined as the proportion of rejection of the null hypothesis for treatment Group 1 based on overall survival. In Scenario 2, both treatment groups were considered effective. Two configurations were examined: $S_{1,D}\left(3\right)=S_{2,D}\left(3\right)=0.6,S_{0,D}\left(3\right)=0.5$, and $S_{1,D}\left(3\right)=0.6,S_{2,D}\left(3\right)=0.55,S_{0,D}\left(3\right)=0.5$. The response rate was fixed at $S_{1,R}\left(0.5\right)=S_{2,R}\left(0.5\right)=0.5$. The power in Scenario 2 was defined as the proportion of rejecting of at least one of the null hypotheses for Group 1 or Group 2 based on overall survival. The number of simulation iterations was set to 10 000 and the number of randomizations in the proposed method was set to 1000.

**TABLE 1:** Simulation scenarios for power evaluation.

	Response rate		3–year survival rate		
	Treatment Group 1	Treatment Group 2	Treatment Group 1	Treatment Group 2	Control (Group 0)
Scenario 1	0.4	0.5	0.6	0.5	0.5
	0.5	0.5	0.6	0.5	0.5
	0.6	0.5	0.6	0.5	0.5
Scenario 2	0.5	0.5	0.6	0.6	0.5
	0.5	0.5	0.6	0.55	0.5

*Note:* Scenario 1 corresponds to a situation in which an overall survival benefit is observed only in treatment Group 1. Scenario 2 corresponds to a situation in which an overall survival benefit is observed in both treatment groups.

### 3.2 Simulation Experiment 2

Simulation studies were conducted to evaluate the performance of the proposed method in a setting with multiple treatment groups to choose from. Out of a total of four groups (g=0,1,2,3), including one control group, up to two treatment groups were selected according to the selection rule defined in Equation (11), with Δ set to 0.05. We assessed the actual type I error rate and power for both the proposed method and the method by Friede et al. The sample size per group in Stage 1 was $N_{g,1}=50$. In Stage 2, the sample size per group $N_{g,2}=204$ or $N_{g,2}=136$, depending on whether one or two treatment groups were selected, respectively. The weights used in the combination test were determined based on the expected number of events. All other conditions, including the enrolment and follow–up periods, were the same as in Simulation Experiment 1.

#### 3.2.1 Type I Error Rate

The settings were similar to those used in Simulation Experiment 1, with $S_{0,D}\left(3\right)=S_{1,D}\left(3\right)=S_{2,D}\left(3\right)=S_{3,D}\left(3\right)=0.5$.

#### 3.2.2 Power

As in Scenario 1 of Simulation Experiment 1, we assumed $S_{1,D}\left(3\right)=0.6,S_{0,D}\left(3\right)=S_{2,D}\left(3\right)=S_{3,D}\left(3\right)=0.5$.

### 3.3 Simulation Experiment 3

In Simulation Experiment 3, we included a conventional phase III fixed design without treatment group selection and compared it with ASD using either the proposed method or the method of Friede et al. Since the number of participants eligible for inclusion in phase III part of the ASD was $N_{g,2}=150$, the total sample size in the phase III fixed design was set to $N_{\mathrm{FD}}=300$, with $N_{\mathrm{FD},g}=100$ per arm. Two pairwise comparisons were conducted at the final analysis. The same two scenarios for power evaluation, as described in Simulation Experiment 1 (Table 1), were used in this experiment.

## 4 Results

### 4.1 Simulation Experiment 1

#### 4.1.1 Type I Error Rate

**FIGURE 1:**
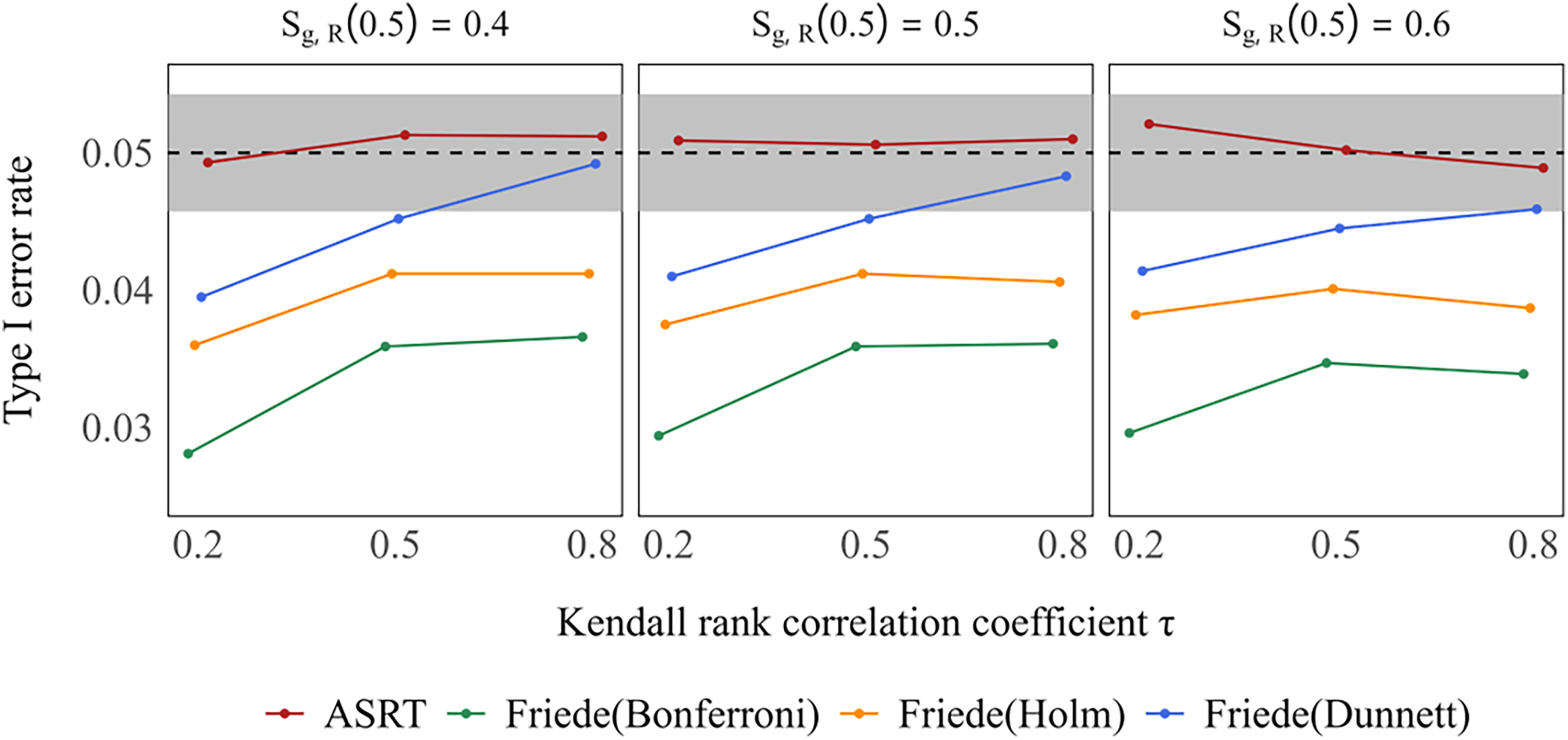
Type I error rate under global null hypothesis (true 3–year survival rates: $S_{0,D}\left(3\right)=S_{1,D}\left(3\right)=S_{2,D}\left(3\right)=0.5$) in case of selecting one treatment group. True response rates were the same across two treatment groups: $S_{1,R}\left(0.5\right)=S_{2,R}\left(0.5\right)=0.4,0.5,0.6.$ The gray shaded area indicates the 95% simulation error band around the nominal 5% significance level.

Figure 1 presents the type I error rates for each method under the global null hypotheses of $S_{0,D}\left(3\right)=S_{1,D}\left(3\right)=S_{2,D}\left(3\right)=0.5$. The method by Friede et al. controlled the type I error rate below the nominal level of 5% across all settings. However, as reported in previous studies, combining the method of Friede et al. with multiple comparison procedures such as the Bonferroni, Holm, and Dunnett methods resulted in more conservative trends that deviated further below the nominal 5% level as the Kendall rank correlation coefficient τ decreased. In contrast, the proposed method exhibited no such conservatism regardless of the value of τ, and the actual type I error rate remained consistently close to the nominal 5% level and the difference was considered to be well within the simulation error (the gray shaded area). Similar results were observed when the response rates were varied. In scenarios where the response rate and overall survival were set to extreme values, similar trends were observed (see Figure S1).

#### 4.1.2 Power

**FIGURE 2:**
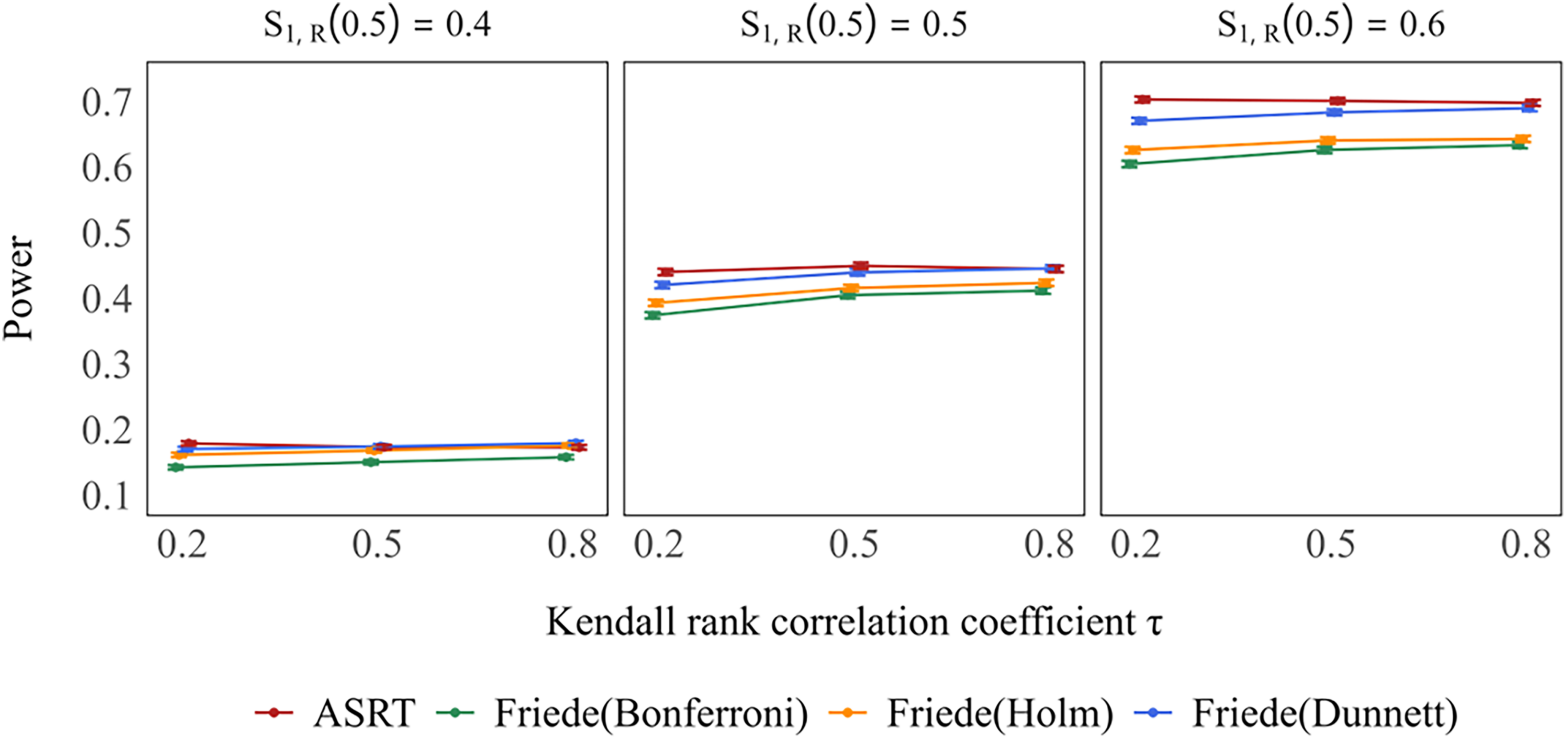
Power of rejecting a false hypothesis for treatment Group 1 under only one treatment group is effective (true 3–year survival rates: $S_{1,D}\left(3\right)=0.6,S_{0,D}\left(3\right)=S_{2,D}\left(3\right)=0.5$) in case of selecting one treatment group. True response rates: $S_{1,R}\left(0.5\right)=0.4,0.5,0.6,\;S_{2,R}\left(0.5\right)=0.5$.

**FIGURE 3:**
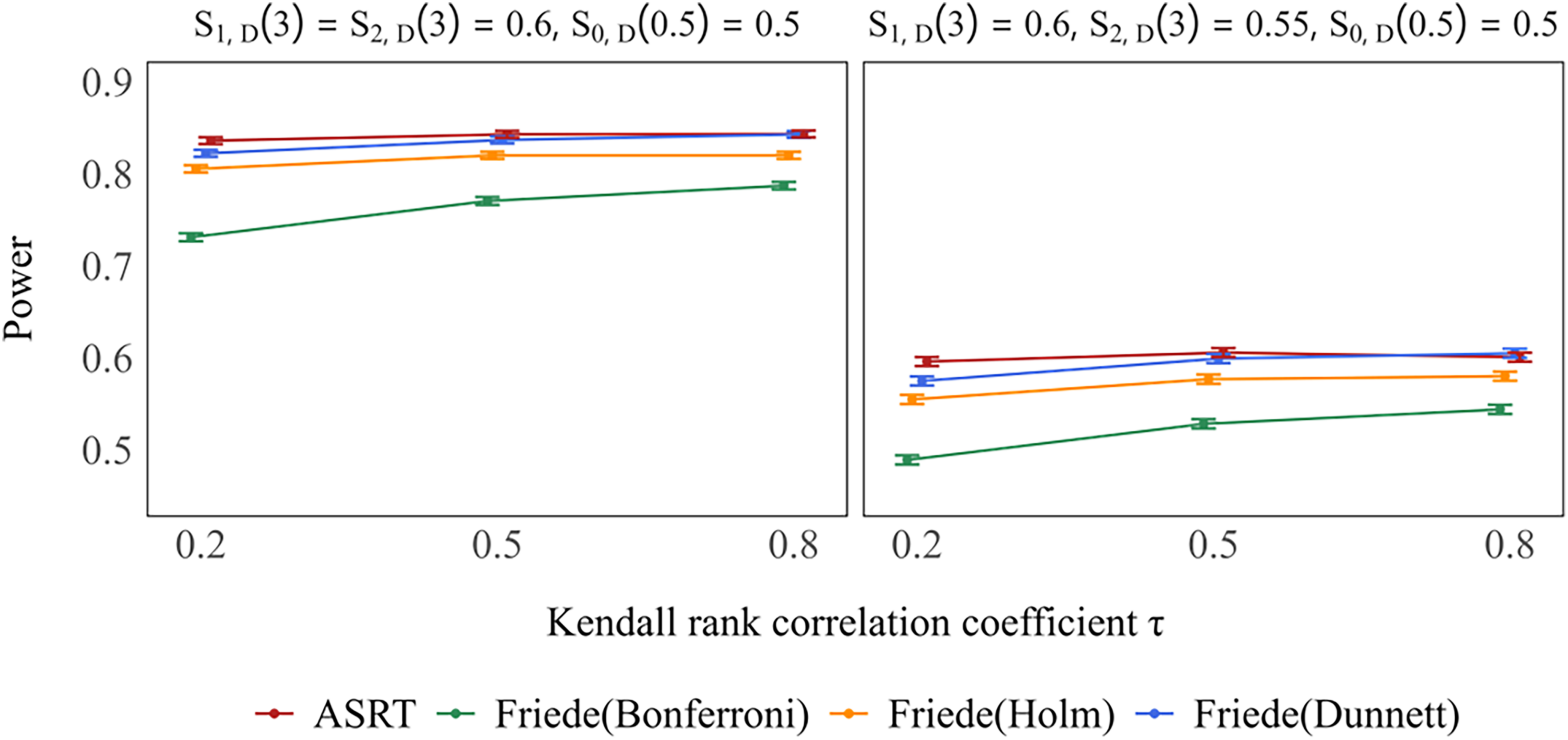
Power of rejecting at least one false hypothesis when both treatment groups are effective is shown in the left panel (true 3–year survival rates: $S_{1,D}\left(3\right)=S_{2,D}\left(3\right)=0.6,\;S_{0,D}\left(3\right)=0.5$), whereas the right panel shows the corresponding power when treatment Group 1 is effective and treatment Group 2 is moderately effective (true 3–year survival rates: $S_{1,D}\left(3\right)=0.6,S_{2,D}\left(3\right)=0.55,S_{0,D}\left(3\right)=0.5$), in case of selecting one treatment group. True response rates: $S_{1,R}\left(0.5\right)=S_{2,R}\left(0.5\right)=0.5.$

Figure 2 shows power when only one treatment group was considered effective. Under the method of Friede et al., power increased as the value of τ increased across all settings. However, the proposed method consistently yielded higher power than the methods of Friede et al., regardless of the value of τ. The actual power also increased with the response rate of treatment Group 1. Among the multiplicity correction methods, Dunnett's method provided the highest power. Figure 3 presents the power when both treatment groups were considered effective. The trends observed for both the method by Friede and the proposed method were similar to those in the single–effectivegroup scenario. Again, the proposed method consistently demonstrated higher power than the method of Friede et al. Even under extreme conditions, the proposed method consistently demonstrated higher power than the method of Friede et al., although the difference between the two methods was reduced (see Figures S2 and S3).

### 4.2 Simulation Experiment 2

#### 4.2.1 Type I Error Rate

**FIGURE 4:**
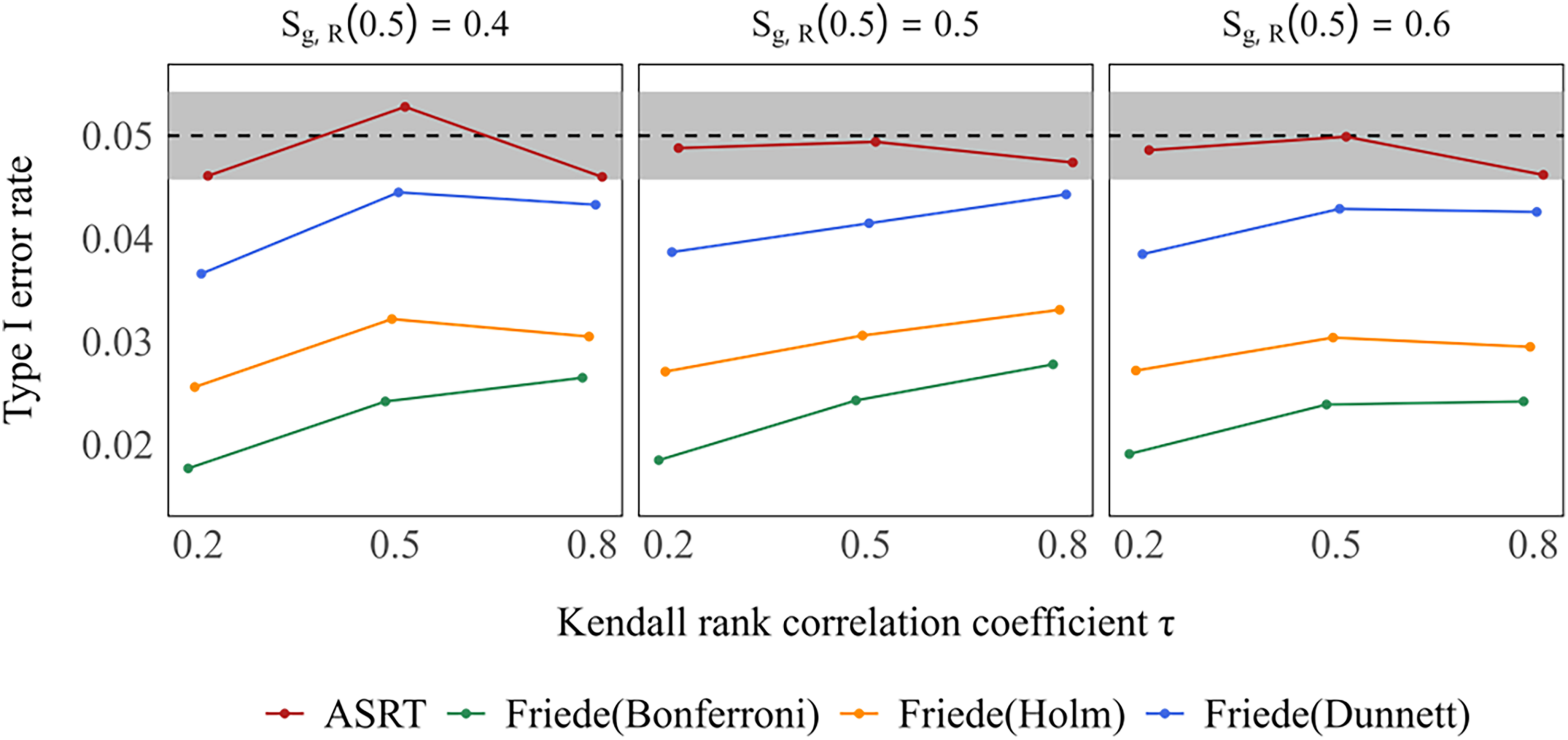
Type I error rate under global null hypothesis (true 3–year survival rates: $S_{0,D}\left(3\right)=S_{1,D}\left(3\right)=S_{2,D}\left(3\right)=S_{3,D}\left(3\right)=0.5$) in case of selecting more than one treatment groups. True response rates were the same across two treatment groups: $S_{1,R}\left(0.5\right)=S_{2,R}\left(0.5\right)=S_{3,R}\left(0.5\right)=0.4,0.5,0.6.$ The gray shaded area indicates the 95% simulation error band around the nominal 5% significance level.

Figure 4 presents the type I error rates for each method under the global null hypotheses of $S_{0,D}\left(3\right)=S_{1,D}\left(3\right)=S_{2,D}\left(3\right)=S_{3,D}\left(3\right)=0.5$ in the setting where more than one treatment group could be selected. The method of Friede et al. controlled the type I error rate below the nominal level 5% in all settings. As in Simulation Experiment 1, the conservative trend was observed as the correlation decreased in almost all cases: all testing methods tended to become more conservative regardless of the degree of correlation. In contrast, the proposed method did not exhibit such conservatism. Across all values of τ, the type I error rate remained consistently close to or just below the nominal level in nearly all scenarios.

#### 4.2.2 Power

**FIGURE 5:**
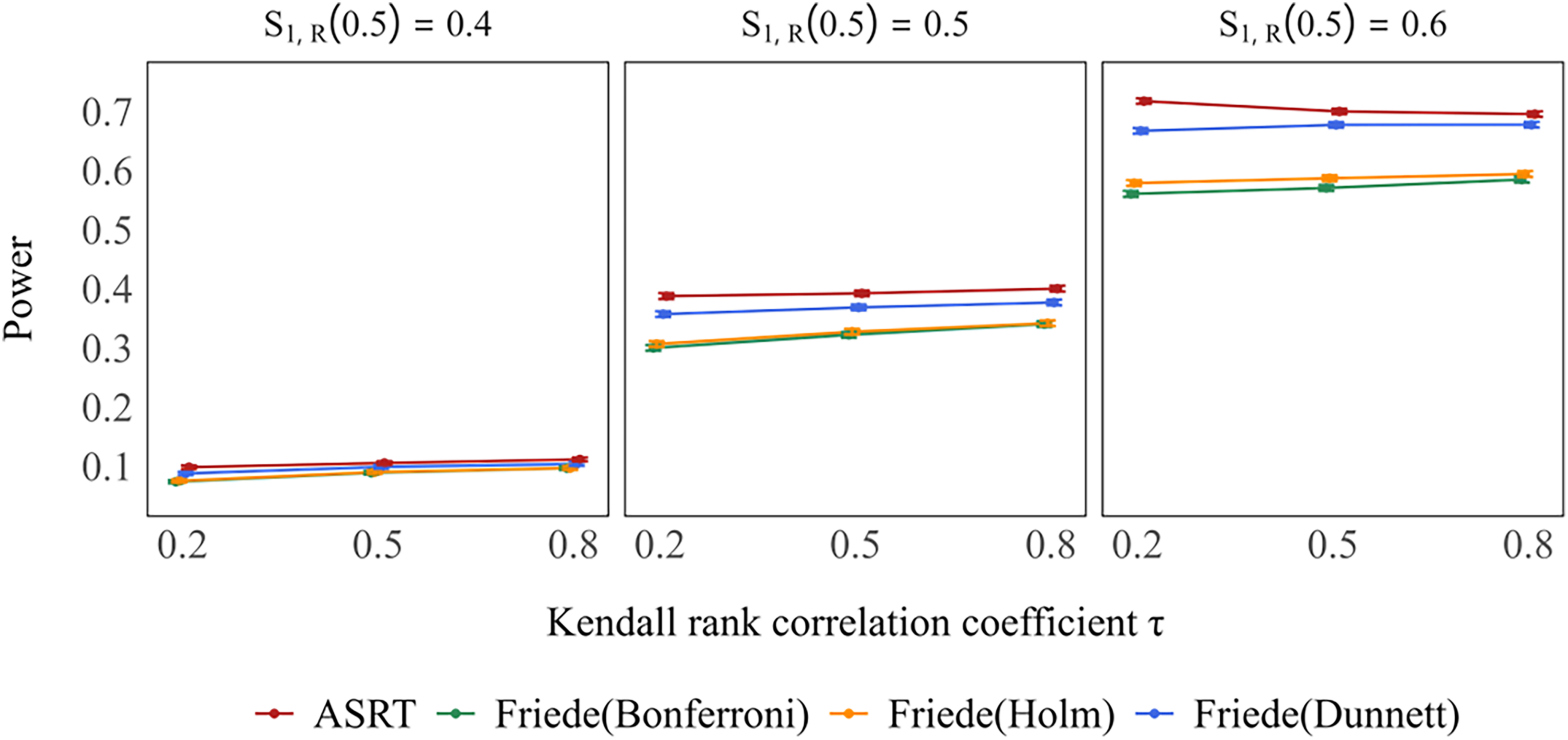
Power of rejecting a false hypothesis for treatment Group 1 under only one treatment group is effective (true 3–year survival rates: $S_{1,D}\left(3\right)=0.6,S_{0,D}\left(3\right)=S_{2,D}\left(3\right)=S_{3,D}\left(3\right)=0.5$) in case of selecting more than one treatment groups. True response rates: $S_{1,R}\left(0.5\right)=0.4,0.5,0.6,\;S_{2,R}\left(0.5\right)=S_{3,R}\left(0.5\right)=0.5$.

Figure 5 shows power when only one treatment group was truly effective $\left(S_{1,D}\left(3\right)=0.6,S_{0,D}\left(3\right)=S_{2,D}\left(3\right)=S_{3,D}\left(3\right)=0.5\right)$ when multiple treatment groups could be selected. The trends for both the Friede method and the proposed method were similar to those observed in the single–group selection setting. The proposed method consistently demonstrated higher power than that proposed of Friede et al. and the better performance was more clearly shown.

### 4.3 Simulation Experiment 3

**FIGURE 6:**
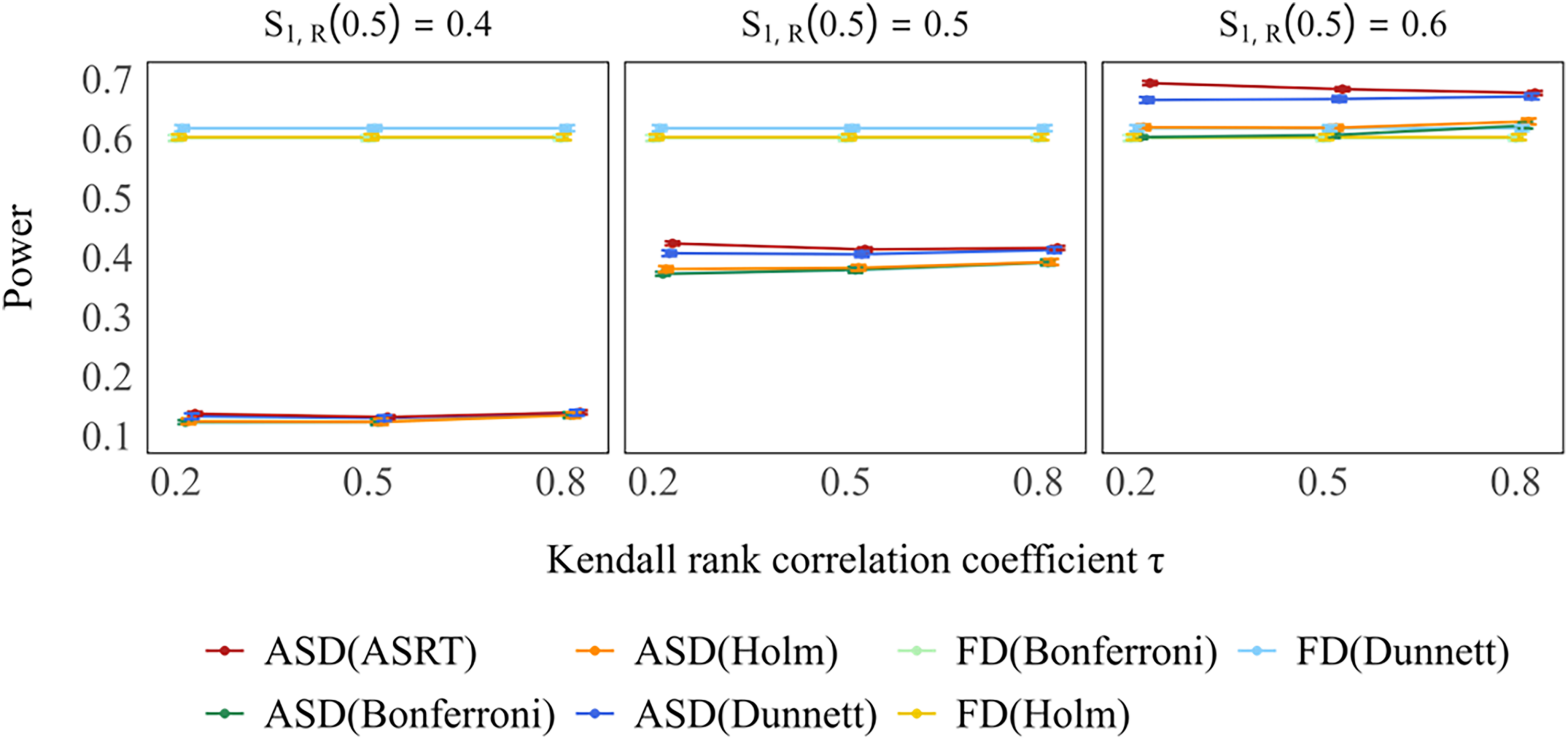
Power for ASD and Fixed Design (FD) under only one treatment group is effective (true 3–year survival rates: $S_{1,D}\left(3\right)=0.6,S_{0,D}\left(3\right)=S_{2,D}\left(3\right)=0.5$) in case of selecting one treatment group. True response rates: $S_{1,R}\left(0.5\right)=0.4,0.5,0.6,\;S_{2,R}\left(0.5\right)=0.5$.

**FIGURE 7:**
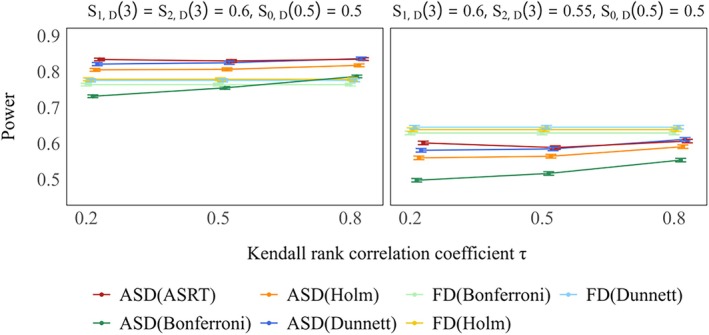
Power for ASD and FD when both treatment groups are effective is shown in the left panel (true 3–year survival rates: $S_{0,D}\left(3\right)=0.5,S_{1,D}\left(3\right)=S_{2,D}\left(3\right)=0.6$), whereas the right panel shows the corresponding power when treatment Group 1 is effective and treatment Group 2 is moderately effective (true 3–year survival rates: $S_{0,D}\left(3\right)=0.5,S_{1,D}\left(3\right)=0.6,S_{2,D}\left(3\right)=0.55$), in case of selecting one treatment group. True response rates: $S_{1,R}\left(0.5\right)=S_{2,R}\left(0.5\right)=0.5$.

Figures 6 and 7 present the power of each design, including the phase III fixed designs, under Scenarios 1 and 2, respectively. In Scenario 1 (only treatment Group 1 is effective), the fixed designs tended to be significantly more powerful than the ASDs when correct selection of treatment Group 1 was difficult. In contrast, ASDs were more powerful than fixed designs when treatment Group 1 could be correctly selected. In Scenario 2 (the treatment Groups 1 and 2 are equally effective), the fixed designs were generally less powerful than the ASDs, as summarized in Table 2. When the effect size for treatment Group 2 was relatively smaller (as shown in Table 3), and the fixed designs were more powerful than the proposed ASD.

**TABLE 2:** Comparison of FD and ASD using the proposed method or the method of Friede et al. (Scenario 1).

τ	Response rate $\left(S_{g,R}\left(0.5\right)\right)$		
	0.4	0.5	0.6
0.2	FD	FD	ASD
0.5	FD	FD	ASD
0.8	FD	FD	ASD

*Note:* Each cell indicates the study design that achieved the highest power. τ denotes the Kendall rank correlation coefficient.

**TABLE 3:** Comparison of FD and ASD using the proposed method or the method of Friede et al. (Scenario 2).

τ	3–year survival rate ($S_{1,D}\left(3\right),S_{2,D}\left(3\right),S_{0,D}\left(3\right)$)	
	0.6, 0.6, 0.5	0.6, 0.55, 0.5
0.2	ASD	FD
0.5	ASD	FD
0.8	ASD	FD

*Note:* Each cell indicates the study design that achieved the highest power. τ denotes the Kendall rank correlation coefficient.

## 5 Discussion

In the development of oncology treatments, a promising treatment group is often selected based on the response rate observed in phase II trials, and the superiority of the selected treatment over the standard treatment is typically evaluated based on overall survival in phase III trials. In this study, we proposed an ASRT tailored to this setting. The proposed test appropriately accounts for the correlation between outcomes based on a randomization distribution. Parametrically modeling the joint distribution of response rate and overall survival and identifying the corresponding correlation structure from the data are generally challenging tasks. By relying on the randomization distribution, the proposed method successfully enabled the accurate calculation of the conditional distribution of the log–rank test statistic for overall survival—conditional on treatment group selection based on the response rate in ASD.

The performance of the proposed method was evaluated though a simulation study modeled after the JCOG2203 trial. The results from Simulation experiment 1 revealed that the proposed method was able to control the type I error rate and correct the conservativeness of the method by Friede et al. [12]. In some cases, the type I error rate slightly exceeded the nominal level; however, this may be due to simulation error, a limited number of randomizations (5000), and the approximation of the log–rank test statistic to the chi–square distribution. Since the log–rank test itself—one of the most widely used methods in practice—is known to exhibit a slight inflation of the type I error rate [30], the deviations observed with the proposed method were considered to be within an acceptable and practically negligible range. Notably, in the setting where multiple treatment groups could be selected, the proposed method was able to control the type I error rate in nearly all scenarios. This is likely because pairwise comparisons occurred in this setting even under the proposed method, and the approach by Friede et al. was applied to address the resulting multiplicity.

The proposed method consistently yielded higher power than the method of Friede et al. across all scenarios. This result may be attributed to the reduced conservativeness with regard to the type I error rate, which in the method of Friede et al. depended heavily on the correlation between outcomes. Indeed, the lower the correlation between the short– and long–term outcomes, the larger the difference in power between the proposed method and that of Friede methods. Specifically, power of the proposed method was approximately 1%–10%, 1%–8%, and 1%–3% higher than that of the Bonferroni, Holm, and Dunnett corrections, respectively, when applied within Friede's framework (Figures 2, 3, and 5). In oncology clinical trials, where patient recruitment is often challenging, the ability to improve power simply by changing the analytical approach represents a significant advantage of the proposed method. Similar results were observed even in the setting where multiple treatment groups were selected. The power of the proposed method was approximately 1%–15%, 1%–14%, and 1%–5% higher than that of the Bonferroni, Holm, and Dunnett correction, respectively, in the method of Friede et al. The better performance was more clearly shown due to further conservatism of multiplicity correction in the method of Friede et al. with more treatment groups considered in phase II part.

In Simulation Experiment 3, we compared ASD with a fixed design without treatment group selection in terms of power. The results showed that ASD outperformed the fixed design when the response rate could identify a truly effective treatment group. These findings can support decision–making regarding whether to adopt ASD or a conventional phase III trial during the planning stage—an essential consideration when implementing ASD. To ensure the effectiveness of treatment group selection at the interim analysis, the expected effect size and variability of short–term outcomes should be carefully evaluated in advance.

This study has several limitations. First, the simulation study considered a three–arm or four–arm trial with two or three experimental treatment groups and one control group and did not consider more than four–arm trial. These settings are based on the JCOG2203 trial and are considered typical configurations in a realistic setting. However, during pharmaceutical development, scenarios involving more than four treatment groups or multiple control groups may arise. Takahashi et al. [16] also conducted a simulation study with three treatment groups. Based on their results, the method of Friede et al. tended to exhibit further conservatism owing to the characteristics of multiple testing adjustments. Our results are also consistent with these results, and these conservatisms would be even stronger for more than four–arm trial. Future studies should also focus on increasing the sample size of a selected group by reallocating resources that would otherwise have gone to the dropped groups if considered in more than four–arm trial. Second, the proposed algorithm did not consider early stopping or sample size reestimation during interim analyses. In oncology clinical trials with long–term follow–up, interim analyses for efficacy or futility are commonly preplanned, especially in phase III trials. These interim assessments, based on overall survival, can be incorporated into the ASD framework by applying group sequential methods with α–spending functions in conjunction with the proposed method. Furthermore, the proposed method can feasibly be extended to incorporate sample size reestimation alongside treatment group selection in ASD, owing to the flexibility of the combination testing approach, which generally allows for modifications to trial design features. Finally, several important considerations for applying the proposed method should be noted. By explicitly defining the selection function, as shown in Equations (4) or (11), the proposed method enables the accurate calculation of the conditional distribution of the log–rank test statistic for overall survival, given treatment group selection and response rates in ASD. Note that the selection function need not be the simplest possible rule one could imagine in applying the proposed method. Our method works with a range of different and quite general rules even considering safety data. In actual clinical trials, treatment groups advancing to phase III are often selected not solely based on efficacy but also on safety profiles derived from a comprehensive assessment. Therefore, selection rules, such as incorporating clinical judgment when efficacy differences fall within a certain margin, should be clearly pre–specified. The assumptions one needs to make if one adopts our approach are that an ASD would comply to the pre–specified selection rule and one can decide whether or not to select the treatment group automatically by applying the pre–specified rule to the rerandomized Stage 1 data. Therefore, the potential impact of deviations from predefined selection rules on the type I error rate should be assessed through simulation studies. For secondary analysis, conservative approaches such as the method of Friede et al., of which validity depends on the closed testing principle and the combination test are not dependent on the pre–specification of selection rules, should be considered. In the case of a deviation from the pre–specified rule due to unexpected circumstances, it would be necessary to make a judgment such as determining significance only when both results are significant. The required assumption of the method of Friede et al. is that the Stage 2 *p*–values are independent of any data used in the selection. Under the assumption, the (asymptotic) *p*–clud condition holds for the combination of each *p*–values, and the FWER is strongly controlled as desired. In situations where one wants to plan an ASD that seamlessly transitions to the next stage in a blinded fashion, rule identification seems essential, but if one wants to leave room for more flexible selection, their method should be chosen. Overall, the application of the proposed method in confirmatory clinical trials requires careful planning, clear pre–specification of selection criteria, and thorough evaluation of potential risks.

Based on the simulation study, the proposed method mitigated the conservatism caused by correlation between short– and long–term outcomes and successfully controlled the type I error rate around the nominal level. Additionally, the power of the proposed method tended to be generally higher than that of the method based on the combination test. Therefore, adopting the proposed method in place of conventional phase III designs is expected to increase the probability of trial success.

## Funding

This work was supported by the Japan Agency for Medical Research and Development (JP21lk0201701).

## Conflicts of Interest

Junki Mizusawa received honoraria from Chugai pharmaceutical, Taiho pharmaceutical; his spouse is an employee of Pfizer. The other authors declare no potential conflicts of interest.

## Supporting information


**Data S1:** sim70400‐sup‐0001‐Supinfo.docx.

## Data Availability

Data sharing not applicable to this article as no datasets were generated or analyzed during the current study.
