# Supplementary material for: Adaptive Seamless Phase II/III Randomization Test Considering Treatment Group Selection Based on Short–Term Binary Outcomes
Source: Stat Med. 2026 Feb 4;45(3-5):e70400. doi: 10.1002/sim.70400 (PMC12871014; doi:10.1002/sim.70400)
Supplement: Supplementary file 1 — Data S1: sim70400‐sup‐0001‐Supinfo.docx. [file SIM-45-0-s001.docx]

Adaptive seamless phase II/III randomization test considering treatment group selection based on short-term binary outcomes

*Supplementary material*


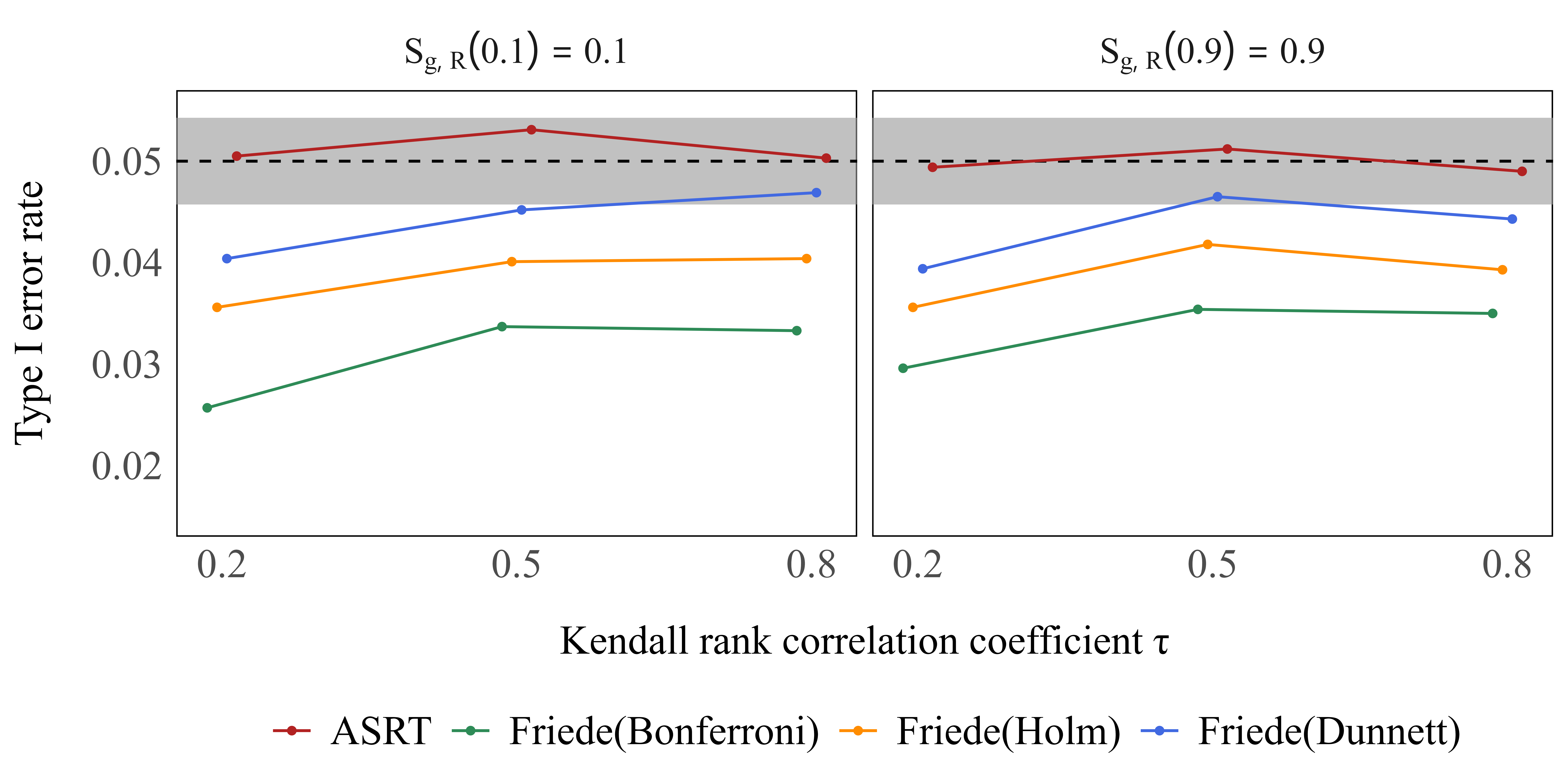


$S_{g, R}\left( 0.5 \right)$: true response rate at the interim analysis in group $g (g=0, 1, 2)$.

**Supplemental Figure 1.** Type I error rate under global null hypothesis in the setting where one treatment group is selected. The left panel shows the results when $S_{0, D}\left( 3 \right)= S_{1, D}\left( 3 \right)=S_{2, D}\left( 3 \right)=0.1$; the right panel shows the results when $S_{0, D}\left( 3 \right)= S_{1, D}\left( 3 \right)=S_{2, D}\left( 3 \right)=0.9$.


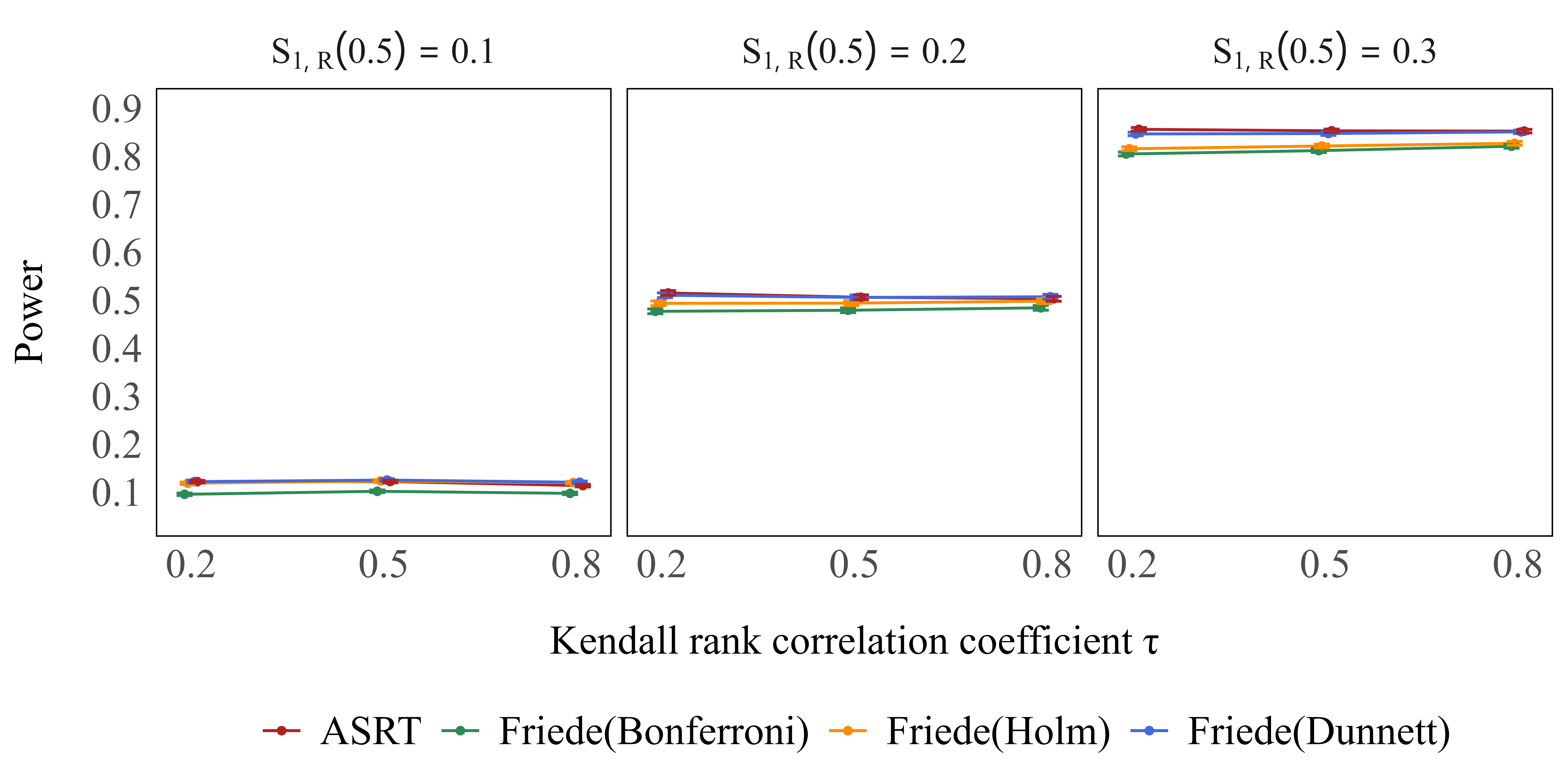


$S_{g, R}\left( 0.5 \right)$: true response rate at the interim analysis in group $g (g=0, 1, 2)$.

**Supplemental Figure 2.** Power of rejecting a false hypothesis for treatment group 1 under only one treatment group is effective $\left( S_{1, D}\left( 3 \right)=0.2, S_{0, D}\left( 3 \right)= S_{2, D}\left( 3 \right)=0.1 \right)$ in case of selecting one treatment group.

**
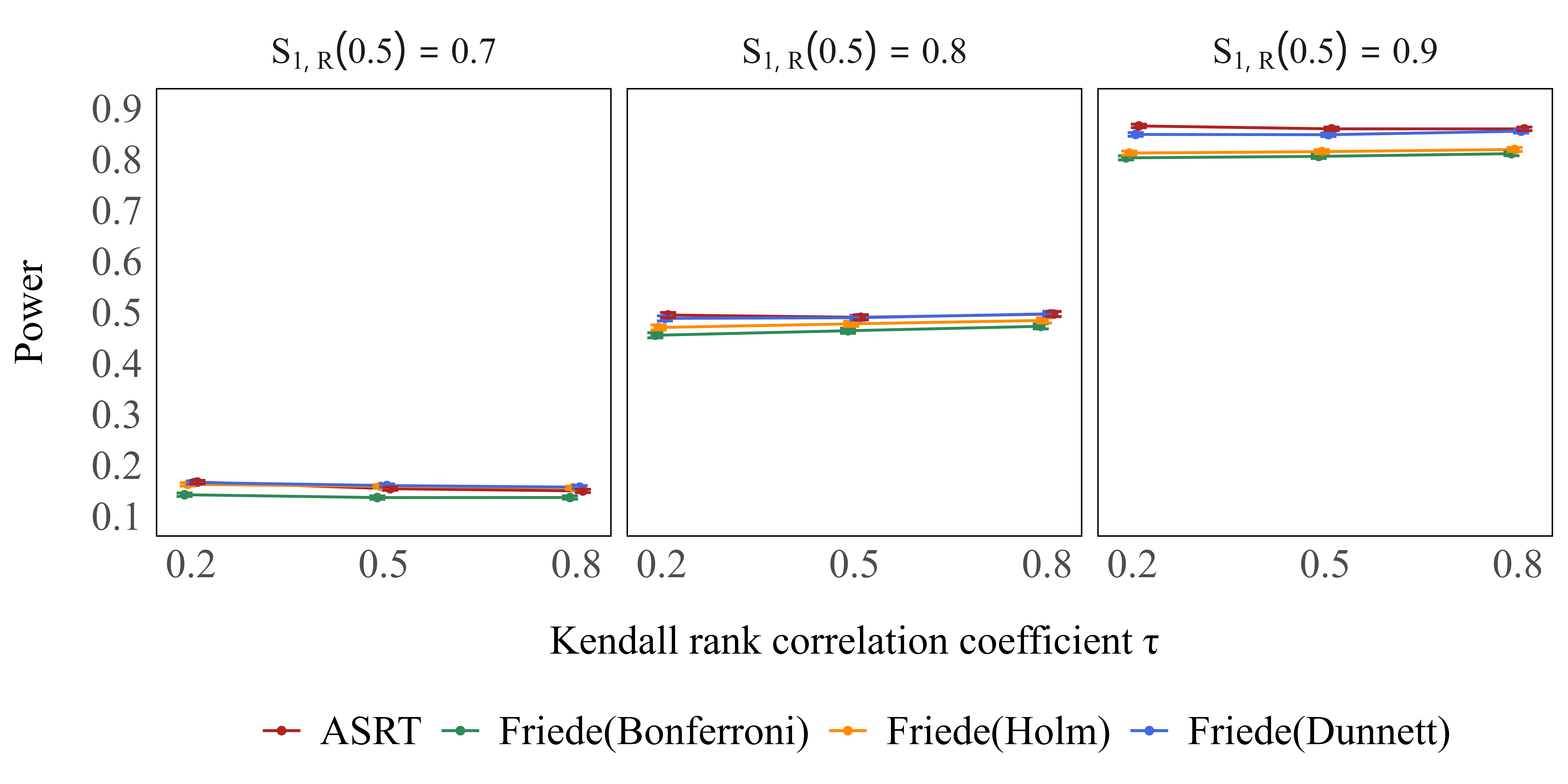
**

$S_{g, R}\left( 0.5 \right)$: true response rate at the interim analysis in group $g (g=0, 1, 2)$.

**Supplemental Figure 3.** Power of rejecting a false hypothesis for treatment group 1 under only one treatment group is effective $\left( S_{1, D}\left( 3 \right)=0.8, S_{0, D}\left( 3 \right)= S_{2, D}\left( 3 \right)=0.7 \right)$ in case of selecting one treatment group.


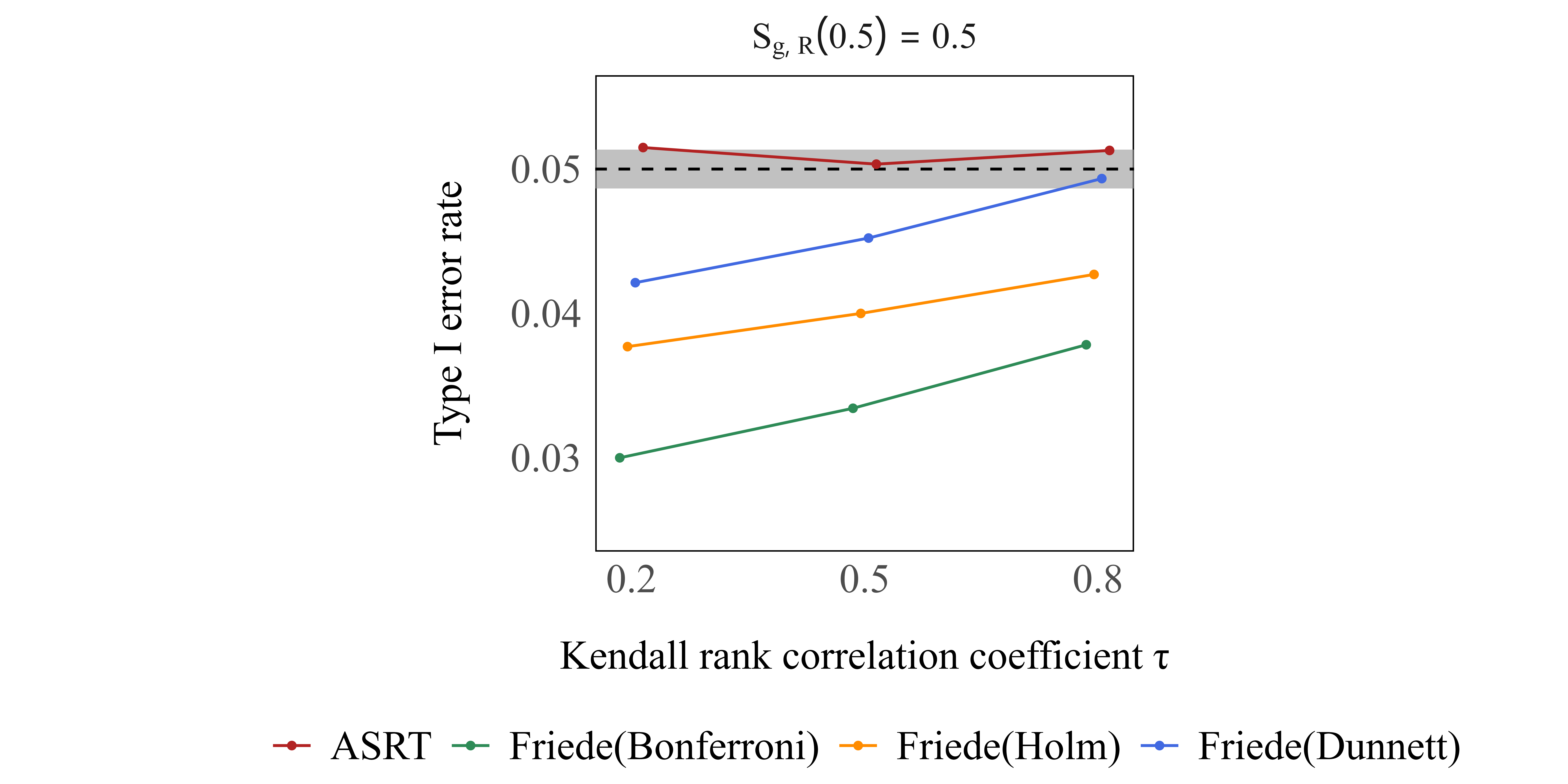


$S_{g, R}\left( 0.5 \right)$: true response rate at the interim analysis in group $g (g=0, 1, 2)$.

**Supplemental Figure 4.** Type I error rate under global null hypothesis $\left( S_{0, D}\left( 3 \right)= S_{1, D}\left( 3 \right)=S_{2, D}\left( 3 \right)=0.5 \right)$ in case of selecting one treatment group, based on 100,000 simulation iterations and 3,000 randomization iterations.
